# *Arx* expansion mutation perturbs cortical development by augmenting apoptosis without activating innate immunity in a mouse model of X-linked infantile spasms syndrome

**DOI:** 10.1242/dmm.042515

**Published:** 2020-03-30

**Authors:** Meagan S. Siehr, Cory A. Massey, Jeffrey L. Noebels

**Affiliations:** 1 Developmental Neurogenetics Laboratory, Department of Neurology, Baylor College of Medicine, One Baylor Plaza, Houston, TX 77030, USA; 2 Molecular and Human Genetics, Baylor College of Medicine, One Baylor Plaza, Houston, TX 77030, USA; 3 Neuroscience, Baylor College of Medicine, One Baylor Plaza, Houston, TX 77030, USA

**Keywords:** Estradiol, ACTH, Interneuronopathy, Inflammation, Epileptic encephalopathy

## Abstract

X-linked infantile spasms syndrome (ISSX) is a clinically devastating developmental epileptic encephalopathy with life-long impact. *Arx^(GCG)10+7^*, a mouse model of the most common triplet-repeat expansion mutation of *ARX*, exhibits neonatal spasms, electrographic phenotypes and abnormal migration of GABAergic interneuron subtypes. Neonatal presymptomatic treatment with 17β-estradiol (E2) in *Arx^(GCG)10+7^* reduces spasms and modifies progression of epilepsy. Cortical pathology during this period, a crucial point for clinical intervention in ISSX, has largely been unexplored, and the pathogenic cellular defects that are targeted by early interventions are unknown. In the first postnatal week, we identified a transient wave of elevated apoptosis in *Arx^(GCG)10+7^* mouse cortex that is non-Arx cell autonomous, since mutant Arx-immunoreactive (Arx^+^) cells are not preferentially impacted by cell death. NeuN^+^ (also known as Rbfox3) survival was also not impacted, suggesting a vulnerable subpopulation in the immature *Arx^(GCG)10+7^* cortex. Inflammatory processes during this period might explain this transient elevation in apoptosis; however, transcriptomic and immunohistochemical profiling of several markers of inflammation revealed no innate immune activation in *Arx^(GCG)10+7^* cortex. Neither neonatal E2 hormone therapy, nor ACTH(1-24), the frontline clinical therapy for ISSX, diminished the augmented apoptosis in *Arx^(GCG)10+7^*, but both rescued neocortical Arx^+^ cell density. Since early E2 treatment effectively prevents seizures in this model, enhanced apoptosis does not solely account for the seizure phenotype, but may contribute to other aberrant brain function in ISSX. However, since both hormone therapies, E2 and ACTH(1-24), elevate the density of cortical Arx^+^-interneurons, their early therapeutic role in other neurological disorders hallmarked by interneuronopathy should be explored.

This article has an associated First Person interview with the first author of the paper.

## Abstract (precis)

**Editor's choice:** We found non-cell-autonomous apoptosis and fewer Arx^+^ interneurons in neonatal neocortex of an X-linked infantile spasms syndrome *Arx* expansion model. Early estradiol therapy rescued Arx^+^ interneuron density, but did not prevent apoptosis.

## INTRODUCTION

Early infantile epileptic encephalopathies (EIEE) include a heterogeneous class of genetic neuronal synchronization disorders hallmarked by their postnatal clinical appearance and complex neurodevelopmental phenotypes (Kalser and Cross, 2018). Mutations in over 20 genes have been identified for EIEE (Axeen and Olson, 2018; McTague et al., 2016), but most molecular details of how and when these single-gene errors perturb the formation of critical brain circuits are not well understood. A clinical subset of the EIEE, the so-called ‘catastrophic epilepsies’, are distinguished by the onset of infantile spasms (IS), developmental cognitive delay and life-long pharmacoresistant epilepsy (Howard and Baraban, 2017). One of the original and best-studied genes in this subgroup is the X-linked transcription factor aristaless-related homeobox (*ARX*) gene (Bienvenu et al., 2002; Colasante et al., 2008). *ARX* mutations are responsible for a rich spectrum of EIEE syndromes, for which phenotypic severity often correlates with the degree of structural damage to the protein, and include X-linked infantile spasms syndrome (ISSX), Ohtahara syndrome and severe dyskinetic disorders (Kato, 2015; Kato et al., 2004; Sherr, 2003; Shoubridge et al., 2010).

Within the forebrain, *Arx* is expressed in developing and mature GABAergic interneurons and is crucial for the migratory capacity of these cells to reach the cortical plate (Colombo et al., 2004; Poirier et al., 2004). This prototypical cell specificity underlying *Arx* pathogenesis inspired the term ‘interneuronopathy’ (Kato and Dobyns, 2005). In addition to controlling migration, the absolute size of the cortical progenitor cell pool in the mouse brain is dependent on *Arx* (Colasante et al., 2015; Friocourt et al., 2008). Given the molecular complexity of *Arx* function, which coordinates the transcription of dozens of known gene targets (Friocourt and Parnavelas, 2011; Fulp et al., 2008; Quillé et al., 2011), much information on the specificity of the cellular pathogenesis underlying *Arx*-related pleiotropy is still missing. Aside from intrinsic deficits in proliferation and migration of Arx^+^ progenitors in the embryonic forebrain, it is not well understood how mutations in *Arx* might impact postnatal processes extrinsic to Arx^+^ interneurons, such as synaptogenesis, cell death and differentiation during early cortical development.

One of the most common mutations in *ARX* is a triplet-repeat expansion in the first polyalanine tract (PA1) that expands this region from ten to 17 alanines (Shoubridge et al., 2010). Patients with this mutation display a less-severe phenotype than individuals with deletion or truncation mutations in *ARX* causing major brain malformations (Kato et al., 2004; Kitamura et al., 2002; Sherr, 2003). Expansion mutations in PA1 alter binding of Arx with transcriptional cofactors and impair transcription regulation, leading to a more subtle disturbance of interneurons (Mattiske et al., 2016; Nasrallah et al., 2012). Mouse models of this expansion have been developed and are viable compared to *Arx* knockout (KO) mice, which show early lethality (Kitamura et al., 2009; Price et al., 2009). Mouse models of PA1 expansion display aberrant migration and loss of several GABAergic interneuron subtypes in the cortex; however, there are differences between models in identity, severity and location of these cellular phenotypes (Beguin et al., 2013; Lee et al., 2017; Price et al., 2009). Mutant *Arx^(GCG)10+7^* males recapitulate human ISSX phenotypes, including neonatal spasms, seizures and behavioral comorbidities, and thus facilitate isolation of *Arx* pathogenic mechanisms (Price et al., 2009). Studies in this model notably reveal interneuronopathy with a selective loss of calbindin^+^ and neuropeptide Y^+^ cells, but sparing parvalbumin^+^ cells (Olivetti et al., 2014; Price et al., 2009).

Complex dysregulation of the Arx-linked transcriptome on survival of distinct neuronal precursor populations remains to be explored. Interestingly, when the effects of *Arx* mutations were examined in pancreatic endocrine progenitors, where *Arx* is expressed during early development, it was found that *Arx* deletion caused aberrant specification of glucagon-producing alpha cells into a beta-islet cell identity, whereas *Arx* expansion mutation led to enhanced apoptosis of alpha cells during development (Wilcox et al., 2013a,b). Since *Arx* expansion causes dysregulation of genes that are directly or indirectly involved in apoptosis (Mattiske et al., 2016), we examined the postnatal *Arx^(GCG)10+7^* mouse brain for inappropriate developmental cell death. Unexpectedly, we identified a transient wave of enhanced programmed cell death in neocortex at the end of the first week that affected only non-Arx^+^ cells. Although we have not positively identified the vulnerable cell type, the onset of this wave coincides with the onset of the spasms phenotype in *Arx^(GCG)10+7^* mice. Since anti-inflammatory hormone treatment with adrenocorticotropic hormone (ACTH) can be clinically effective in reducing infantile spasms (Song et al., 2017), we looked for evidence of cellular inflammation that might account for the enhanced neonatal cell death, but found none. We also determined that the anti-epileptogenic efficacy of the neuroprotective hormone, 17β-estradiol (E2) in this model, as well as ACTH peptide fragment 1-24 [ACTH(1-24)], is likely due to rescue of Arx^+^ interneurons rather than mitigating the wave of elevated cortical apoptosis.

## RESULTS

### *Arx^(GCG)10+7^* mutants exhibit an enhanced wave of CC3-mediated apoptosis in the neocortex during the first postnatal week

**Fig. 1.**
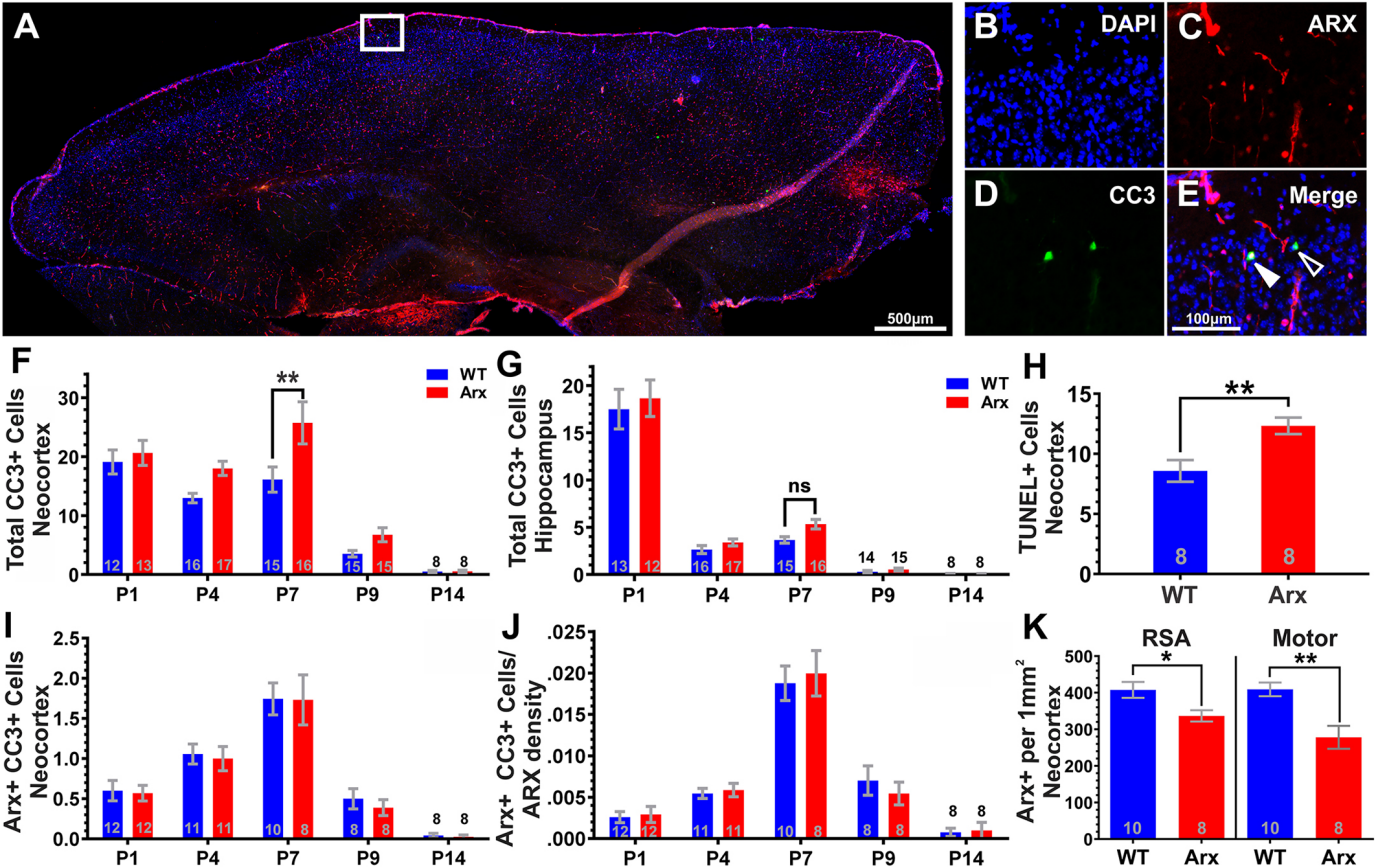
***Arx^(GCG)10+7^* expansion mutants exhibit age-dependent increase in non-cell-autonomous apoptosis.** (A-E) Representative images of a parasagittal cortical section of P7 *Arx^(GCG)10+7^* showing CC3-immunoreactive (CC3^+^) (green) and Arx-immunoreactive (Arx^+^) (red) cells in the neocortex. (B-E) Enlarged images of the white box in A, showing DAPI (B), Arx (C), CC3 (D) and merged (E) immunostaining. (E) Open arrowhead shows a CC3^+^ cell and filled arrowhead shows a CC3^+^ cell with Arx^+^ nucleus. (F,G) Histograms comparing mean CC3^+^ cells at P1, P4, P7, P9 and P14 from cortices of WT and *Arx^(GCG)10+7^* littermates (*N*=8-15 WT, *N*=8-16 mutant) in neocortex (F) and hippocampus (G). (F) In neocortex, two-way ANOVA yields an effect of genotype [*F*(1,119)=5.338, *P*=0.0025]. (G) In hippocampus, two-way ANOVA yields no effect of genotype [*F*(1,72)=0.9080, *P*=0.2035]. (H) Mean fluorescein isothiocyanate (FITC)^+^ (TUNEL^+^) cells in neocortex from TUNEL assay at P7 (*P*=0.0051, unpaired Student's *t*-test, *N*=8 WT, 8 Arx). (I) Histograms comparing mean CC3^+^ cells that co-express Arx (Arx^+^ CC3^+^). Two-way ANOVA yields no effect of genotype [*F*(1,88)=0.2374, *P*=0.6273] (*N*=8-12 WT, 8-12 mutant). (J) Histograms comparing mean co-labeled (CC3^+^ Arx^+^) cells normalized to Arx^+^ density. Two-way ANOVA yields no effect of genotype [*F*(1,88)=0.01996, *P*=0.8880] (*N*=8-12 WT, 8-12 mutant). (K) Mean Arx^+^ density (Arx^+^ cells/mm^2^) at P7 in the retrosplenial agranular region (RSA) (*P*=0.0225) and motor cortex (*P*=0.0017) (unpaired Student's *t*-test) of *Arx^(GCG)10+7^* mutants compared to WT. F-G and I-J utilized two-way ANOVA with Bonferroni's multiple comparisons test. Scale bars: 500 µm (A) and 100 µm (E), with images in B-E scaled similarly. Means±s.e.m. are displayed, with exact *N* values displayed on graphs.*P<0.05, **P<0.01. ns, not significant.

To test the hypothesis that *Arx^(GCG)10+7^* expansion mutation may alter patterns of apoptosis in the postnatal brain, we examined *Arx^(GCG)10+7^* and wild-type (WT) littermates using a specific antibody against cleaved caspase-3 (CC3; also known as Casp3), a marker of the execution phase of apoptosis (Porter and Jänicke, 1999). We profiled CC3-expressing cells at five time points during the first 2 weeks of life [postnatal day (P)1, P4, P7, P9 and P14] across the rostrocaudal extent of the neocortex and hippocampus in parasagittal brain sections of mutant *Arx^(GCG)10+7^* pups and WT littermates (Fig. 1A-E). Two-way ANOVA yielded a significant effect of genotype on CC3-immunoreactive (CC3^+^) levels in the neocortex (*P*=0.0025, Fig. 1F), and Bonferroni's multiple comparisons test yielded a significant, 25-35% increase in CC3^+^ cells in *Arx^(GCG)10+7^* neocortex compared to WT littermates at P7 (*P*=0.0012, Fig. 1F). Binning analysis in the neocortex revealed that CC3^+^ cell localization did not differ between genotypes in terms of laminar position (Fig. S1A). In contrast, CC3^+^ cells were rarely present in the hippocampus after P1. When found, they were typically in the CA1 region; however, there was no significant effect of genotype (*P*=0.2035, two-way ANOVA) and no significant differences between genotypes at any age in the hippocampus (Fig. 1G). The elevated apoptosis in P7 neocortex was confirmed by terminal deoxynucleotidyl transferase dUTP nick end labeling (TUNEL) assay, which showed an overall increase in TUNEL-labeled cells at P7 in mutants (*P*=0.0051, unpaired Student's *t*-test, Fig. 1H). Overall, these data demonstrate a transient increase in CC3^+^ cells in the neocortex of mutants, but not hippocampus, with the most robust difference occurring at P7.

### Mutant Arx^+^ cortical interneurons do not exhibit altered apoptosis

We initially expected that the augmented cell death was likely cell autonomous and that the *Arx^(GCG)10+7^* mutation enhanced apoptosis in this interneuron population. During the first two postnatal weeks, GABAergic interneurons normally undergo a wave of apoptosis, reducing their population density by ∼20-30%, with a peak in apoptosis at P7 (Priya et al., 2018; Southwell et al., 2012). We therefore predicted that the increase in CC3^+^ cells in the mutant is due to elevated death of a subpopulation of Arx-immunoreactive (Arx^+^) interneurons expressing the expansion mutation, similar to its effects in pancreas (Wilcox et al., 2013a). To test this, we profiled the developing cortex using parasagittal brain sections and specific antibodies against Arx and CC3 and looked for an increase in Arx^+^ cells that express CC3. However, at all ages profiled (P1, P4, P7, P9 and P14), counts of Arx^+^ and CC3^+^ co-labeled cells did not significantly differ between *Arx^(GCG)10+7^* and WT littermate control brains [*F*(1,88)=0.2374, *P*=0.6273, two-way ANOVA, Fig. 1I]. Since the *Arx^(GCG)10+7^* neocortex exhibited a reduced Arx^+^ cell density (Fig. 1K) (Price et al., 2009), there may be proportionally more Arx^+^ cells labeled with CC3 in *Arx^(GCG)10+7^* mutants compared to WT. However, normalizing co-labeled Arx^+^ CC3^+^ cells to Arx^+^ density counts at each age did not reveal any significant differences between genotypes [*F*(1, 88)=0.01996, *P*=0.8880, Fig. 1J]. These data indicate that the increased apoptosis present in *Arx^(GCG)10+7^* mutant cortex is extrinsic, i.e. non-cell autonomous, as it does not reflect a concomitant increase in Arx^+^ cell death.

### *Arx^(GCG)10+7^* does not impact survival of NeuN-expressing cortical neurons

**Fig. 2.**
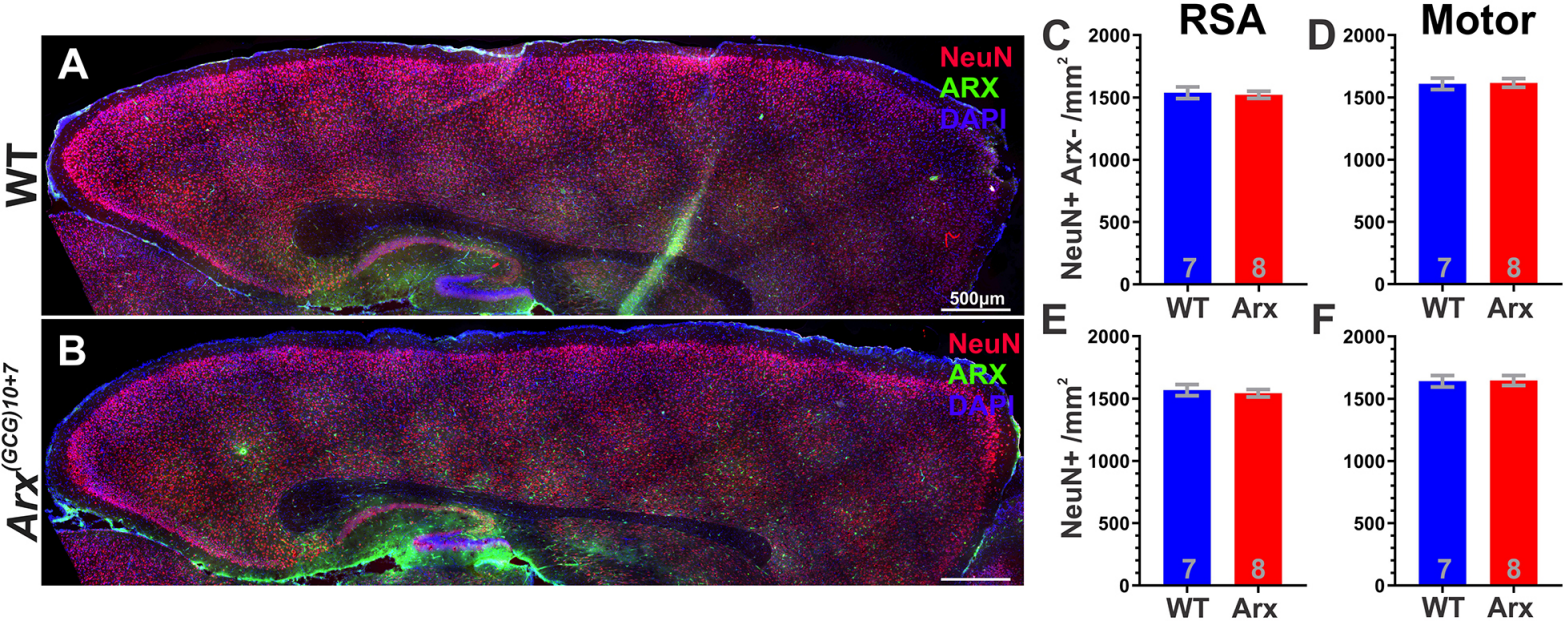
***Arx^(GCG)10+7^* expansion mutants do not exhibit loss of Arx^−^** **cortical neurons at P14.** (A,B) Representative images of WT (A) and *Arx^(GCG)10+7^* (B) parasaggital sections showing NeuN- and Arx-immunoreactive cells. (C,D) Mean density of NeuN^+^ cells that do not express Arx (NeuN^+^ Arx^−^/mm^2^) in two neocortical regions: RSA (*P*=0.7575, unpaired Student's *t*-test) (C) and motor cortex (*P*=0.8665, Mann–Whitney test) (D). (E,F) Mean total NeuN density (NeuN^+^ cells/mm^2^) of two neocortical regions: RSA (*P*=0.6511, unpaired Student's *t*-test) (E) and motor cortex (*P*=0.9551, Mann–Whitney test) (F). Scale bars: 500 µm. Means±s.e.m. are displayed. Exact *N* values are displayed on graphs.

Since *Arx^(GCG)10+7^* does not affect the neonatal survival of Arx^+^ interneurons, other cells must account for elevated neonatal CC3^+^ expression. During embryonic development, *Arx* is transiently expressed in neural progenitors that also give rise to cortical excitatory neurons; however, these neurons do not retain *Arx* expression upon exiting the subventricular niche (Colasante et al., 2008; Colombo et al., 2004; Friocourt et al., 2006). Therefore, we speculated that Arx^(GCG)10+7^ mutant protein might alter transcriptional networks in this progenitor subpopulation and affect their postnatal survival despite the absence of mutant Arx. If apoptosis within this population is increased at P7, we might expect to observe a concomitant reduction in cortical neuron density at a later age. To test this, we profiled neuronal survival 1 week later (P14) in the cortex of *Arx^(GCG)10+7^* and WT littermates using antibodies against NeuN (also known as Rbfox3) and Arx. NeuN is a pan-neuronal marker, whereas Arx is expressed in ∼70-90% of GABAergic interneurons, depending on brain and cortical region (Colombo et al., 2004; Poirier et al., 2004). Therefore, a majority of NeuN neurons that do not express Arx (NeuN^+^ Arx^−^) are cortical excitatory neurons. We quantified the densities of NeuN^+^ Arx^−^ and total NeuN density in two neocortical regions: the retrosplenial agranular region (RSA) and motor cortex in P14 parasagittal slices (Fig. 2A,B). We found no difference in the densities of NeuN^+^ neurons that do not express Arx (NeuN^+^ Arx^−^/mm^2^) in the RSA (*P*=0.7575, unpaired Student's *t*-test, Fig. 2C) or in motor cortex (*P*=0.8665, Mann–Whitney test, Fig. 2D) at this age. We also profiled total NeuN density (NeuN^+^/mm^2^) and found no significant differences (RSA, *P*=0.6511, unpaired Student's *t*-test, Fig. 2E; motor cortex, *P*=0.9551, Mann–Whitney test, Fig. 2F). These results suggest that *Arx^(GCG)10+7^* does not affect the survival of cortical non-Arx-expressing neurons.

### Innate immune response does not account for enhanced neonatal apoptosis in *Arx^(GCG)10+7^* cortex

**Fig. 3.**
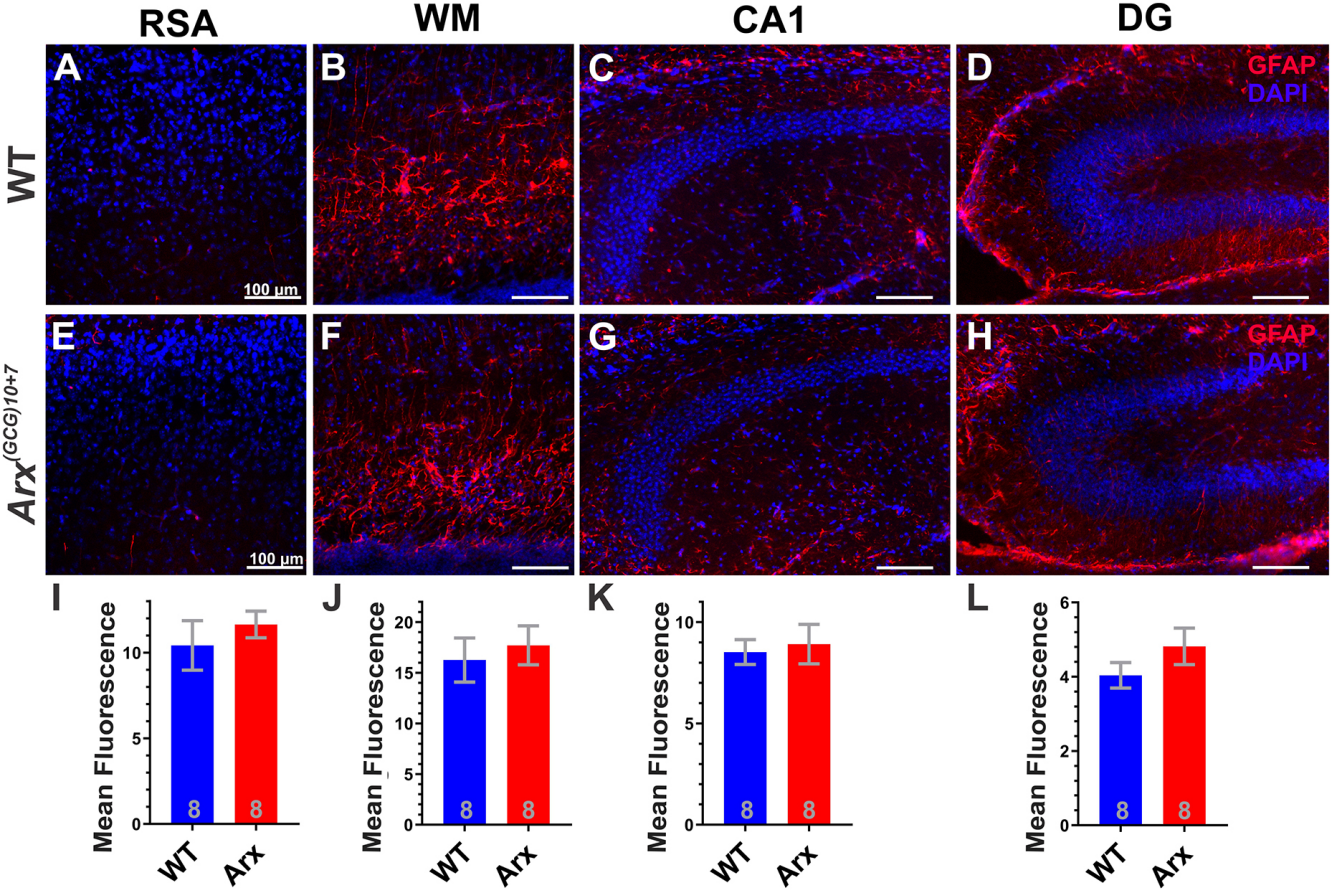
***Arx^(GCG)10+7^* expansion mutants do not exhibit an astrocytic response at P7.** (A-H) Representative images from parasagittal slices showing GFAP immunohistochemistry (red) and DAPI nuclei (blue) from WT (A-D) and *Arx^(GCG)10+7^* (E-H) littermates. Images show each region quantified: RSA (A,E), white matter (WM; B,F), CA1 of the hippocampus (CA1; C,G) and dentate gyrus of the hippocampus (DG; D,H). (I-L) Mean fluorescence quantified from each region specified and graphs display average values for each genotype in the RSA (*P*=0.4686, unpaired Student's *t*-test) (I), WM (*P*=0.6277, unpaired Student's *t*-test) (J), CA1 (*P*=0.7385, unpaired Student's *t*-test) (K) or DG (*P*=0.2139, unpaired Student's *t*-test) (L). *N*=8 WT, *N*=8 mutant. Scale bars: 100 µm. Means±s.e.m. are displayed, with *N* values displayed on graphs.

The increased CC3^+^ cell number in mutant neocortex is temporally confined to the first week of life and cannot be attributed to increased cell death of mutant Arx^+^ interneurons or reduced survival of NeuN^+^ neurons. Acute and chronic inflammation in the immature brain can lead to apoptosis (Hagberg et al., 2015) and inflammation is a known cause of IS (Shandra et al., 2017). Thus, we speculated that an activated innate immune response in the postnatal *Arx^(GCG)10+7^* brain might contribute to increased cell death, and, if so, could provide a basis for neuroactive hormone therapy. We therefore examined immunohistochemical markers of inflammation including astrogliosis and microgliosis and profiled inflammatory cytokine mRNA expression in cortical samples using Nanostring and quantitative PCR (qPCR). We found no significant differences between *Arx^(GCG)10+7^* and WT brain in astroglial activation via GFAP immunoreactivity at P7, the age of maximal CC3 expression (Fig. 3). Since this antibody was inadequate for accurate cell body counting, total GFAP antibody fluorescence was quantified in several brain regions at P7, and these regions did not exhibit any apparent microscopic differences in astrocyte morphology (Fig. 3A-H) nor any significant differences in mean GFAP fluorescence intensity (Fig. 3I-L). Regions profiled included the RSA of the neocortex (*P*=0.4686, unpaired Student's *t*-test, Fig. 3I), white matter (*P*=0.6277, unpaired Student's *t*-test, Fig. 3L), and the CA1 (*P*=0.7385, unpaired Student's *t*-test, Fig. 3K) and dentate gyrus (*P*=0.2139, unpaired Student's *t*-test, Fig. 3L) of the hippocampus.

**Fig. 4.**
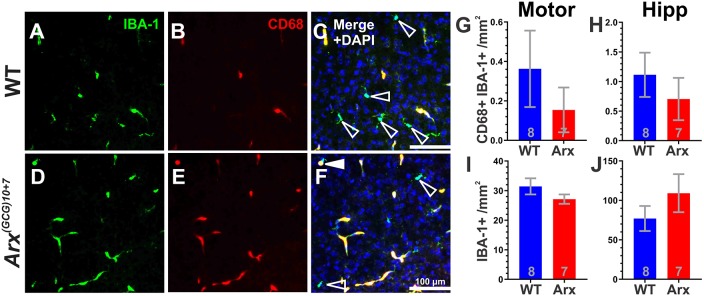
***Arx^(GCG)10+7^* expansion mutants do not exhibit microglial invasion or activation in the forebrain at P7.** (A-F) Representative images of WT (A-C) and *Arx^(GCG)10+7^* (D-F) neocortex at P7 showing IBA-1 (A,D) and CD68 (B,E) immunoreactivity, and merged images plus DAPI (C,F). Open arrowheads indicate IBA-1^+^ cells and filled arrowhead indicates a IBA-1^+^ cell with CD68^+^ nucleus. (G,H) Mean IBA-1^+^ cells that are CD68 immunoreactive per mm^2^ in the motor cortex (Motor) (*P*=0.5562, Mann–Whitney test) (G) and hippocampus (Hipp) (*P*=0.3260, Mann–Whitney test) (H). (I,J) Mean IBA-1^+^ cells per mm^2^ in the motor cortex (Motor) (*P*=0.2093, unpaired Student's *t*-test) (I) and hippocampus (Hipp) (*P*=0.2748, unpaired Student's *t*-test) (J). *N*=8 WT, *N*=7 *Arx^(GCG)10+7^*. Scale bars: 100 µm. Means±s.e.m. are displayed; exact *N* values are displayed on graphs.

**Fig. 5.**
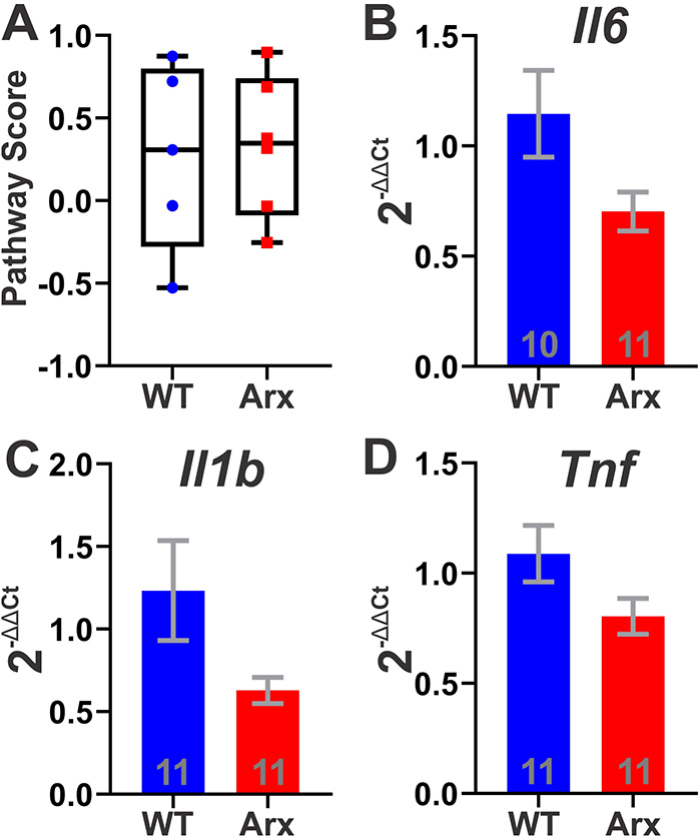
***Arx^(GCG)10+7^* mutation does not affect brain cytokine mRNA expression.** (A) Box plot of cytokine pathway scores generated from a group of 50 cytokine and cytokine-related mRNAs using the first principal component of their expression data generated from Nanostring nCounter(R) Neuropathology Plus Panel (*P*=0.3095, Mann–Whitney test; *N*=5 WT, *N*=6 Arx). Whiskers denote minimum and maximum values; box plot shows median with 25th and 75th percentiles. (B-D) Average 2^−ΔΔCt^ for mRNA expression of cytokines *Il1b*, *Il6* and *Tnf* at P7 using *Lars* expression for normalization: *Il6* (*P*=0.1321, Mann–Whitney test) (B), *Il1b* (*P*=0.0652, Mann–Whitney test) (C) and *Tnf* (*P*=0.1513, Mann–Whitney test) (D). Means±s.e.m. are displayed; *N* values are displayed on graphs.

Similarly, microglial activation was profiled using antibodies against IBA-1 and CD68 (Fig. 4A-F). IBA-1 is a microglial marker and CD68 is expressed by microglia that are actively phagocytic (Witcher et al., 2015). There were no significant differences in IBA-1^+^ CD68^+^ cell densities between genotypes in the motor cortex (*P*=0.3889, unpaired Student's *t*-test) or hippocampus (*P*=0.3260, Mann–Whitney test) (Fig. 4G,H), nor any significant differences in IBA-1^+^ cell densities in the motor cortex (*P*=0.2093, unpaired Student's *t*-test) or hippocampus (*P*=0.3889, unpaired Student's *t*-test) (Fig. 4I,J), indicating no changes in the level of microglial activation and invasion between genotypes, respectively. This lack of evidence of cellular inflammatory activity was supported by cortical mRNA expression profiling studies, which did not reveal any significant differences in cytokine mRNA levels according to Nanostring Pathway Scoring on a total of 50 cytokines and cytokine-related genes (*P*=0.9307, Mann–Whitney test, Fig. 5A; see Table S1 for gene list). In addition, qPCR validation of canonical neuroinflammatory cytokines (*Il1b*, *Il6* and *Tnf*) did not indicate changes in cortical mRNA expression between P7 *Arx^(GCG)10+7^* and WT littermates (Fig. 5B-D) (*Il1b*, *P*=0.0652, Mann–Whitney test; *Il6*, *P*=0.1321, Mann–Whitney test; *Tnf*, *P*=0.1513, Mann–Whitney test). Taken together, these findings indicate that the transient increase in neonatal cortical apoptosis in *Arx^(GCG)10+7^* mutants at P7 is unlikely to be driven by an inflammatory process.

### Early hormone therapies modulate interneuron density but not apoptosis in neonatal *Arx^(GCG)10+7^* mice

**Fig. 6.**
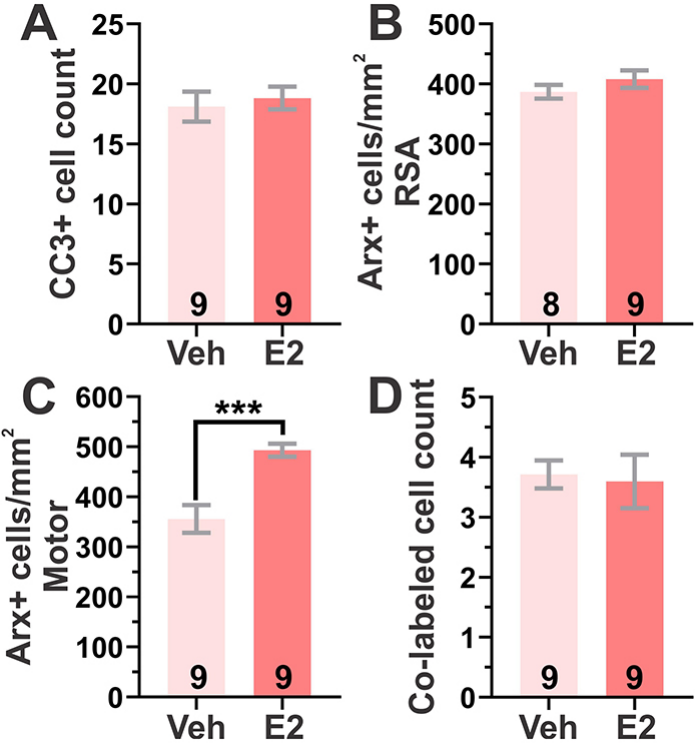
**E2 treatment modifies neonatal cellular pathology in *Arx^(GCG)10+7^*.** (A-C) Effect of P3-P7 40 µg/kg/day E2 treatment compared to vehicle (Veh) treatment (sesame oil) on CC3^+^ cell counts and Arx^+^ cell density in *Arx^(GCG)10+7^* at P7. (A) Mean CC3^+^ counts in neocortex with E2 treatment (*P*=0.6548; unpaired Student's *t*-test). (B,C) Mean Arx^+^ cell density with E2 treatment in RSA (*P*=0.2804, unpaired Student's *t*-test) (B) and motor cortex (*P*=0.0004, unpaired Student's *t*-test) (C). (D) Mean neocortical Arx^+^ CC3^+^ co-labeled cell counts with E2 treatment (*P*=0.8177, unpaired Student's *t*-test). ***P<0.001. Means±s.e.m. are displayed. All *N* values are displayed on graphs.

We next sought to determine whether pretreatment with E2, a multifaceted, estrogen receptor-dependent (and independent) neuroactive hormone (Azcoitia et al., 2019), might ameliorate the elevated CC3-mediated cortical apoptosis in *Arx^(GCG)10+7^*, thus providing a mechanism for its early protective effect. Previously, we showed that E2 treatment reduces neonatal spasms and aberrant electrographic phenotypes including seizures and interictal discharge in adults when administered daily during the first week of life, but not later in adult *Arx^(GCG)10+7^* mutants (Olivetti et al., 2014). Thus, the increase we observed in CC3 expression at P7 overlaps with the effective time for E2 treatment (P3-P10). We treated mutant cohorts with E2 (40 µg/kg/day) or vehicle (sesame oil) daily from P3 to P7 and evaluated cortical cell death at P7. E2 had no effect on cortical CC3^+^ counts (*P*=0.6548, unpaired Student's *t*-test, Fig. 6A). In addition, we quantified Arx^+^ cell density in two cortical areas, the RSA and motor cortex, following treatment. E2 had no effect on P7 Arx^+^ cell density in the RSA (*P*=0.2804, unpaired Student's *t*-test, Fig. 6B); however, E2 significantly increased Arx^+^ cell density in the motor cortex (*P*=0.0004, unpaired Student's *t*-test, Fig. 6C). E2 rescued Arx^+^ densities similar to WT levels in the motor cortex with a mean of 409.2±59.60 Arx^+^ cells/mm^2^ in WT (Fig. 1K) and 493.2±38.8 Arx^+^ cells/mm^2^ in E2-treated *Arx^(GCG)10+7^* (Fig. 6C). We also determined that P3-P7 E2 treatment did not affect the number of Arx^+^ cells undergoing apoptosis at P7, as the number of Arx^+^ cells that expressed the CC3 apoptotic marker was similar between hormone- and vehicle-treated controls (*P*=0.8177, unpaired Student's *t*-test, Fig. 6D), suggesting that the mechanism by which E2 increases Arx^+^ cell density is not by improving their survival. Similarly, we examined the effect of ACTH(1-24), a widely used clinical therapy for IS (Kelley and Knupp, 2018; Song et al., 2017). We first tested the efficacy of early ACTH(1-24) treatment on aberrant phenotypes in *Arx^(GCG)10+7^* and found that ACTH(1-24) was ineffective in this model at two different doses (Fig. S2A-E). Interestingly, ACTH(1-24)-treated mutants exhibited an elevated number of cortical CC3^+^ cells compared to vehicle control (Fig. S2F), yet similar to E2 treatment, ACTH(1-24) increased Arx^+^ cell density in the motor cortex but not the RSA (Fig. S2G,H).

## DISCUSSION

Deletions or truncating mutations in *ARX* result in major human cortical malformations and early lethality in *Arx* mouse models (Kato et al., 2004; Kitamura et al., 2002). A more subtle disturbance of this transcription factor in both human and mouse models leads to serious neurodevelopmental disease with only microscopic evidence of interneuron migration failure (Kitamura et al., 2009; Lee et al., 2017; Price et al., 2009), which offers an opportunity to isolate early pathogenic mechanisms before misleading terminal stages. Studies on *Arx* have focused on identifying its transcriptional targets and their role in embryonic brain development and adult pathophysiology; however, less is known about their impact upon programmed cell death during the early neonatal period of cortical maturation, a point at which detrimental phenotypes such as IS appear and clinical intervention is crucial. In this study, we investigated how the *Arx^(GCG)10+7^* expansion mutation, a humanized genetic model of ISSX with impaired cellular migration, disrupts programmed cell death and whether established treatments have an effect on this neonatal cellular endophenotype. We found an enhancement of extrinsic apoptosis in the early postnatal brain that may alter the balance of network excitability, thereby contributing to epilepsy and other developmental comorbidities. However, neither early E2 treatment, which is effective in preventing seizures in this model, nor ACTH, a clinically effective treatment in some cases of IS, mitigated elevated cell death, indicating that these treatments have a complex relationship with the neurodevelopmental phenotype in *Arx^(GCG)10+7^*.

### Identity of non-cell-autonomous apoptosis in *Arx^(GCG)10+7^*

In WT prenatal brain, *Arx* is expressed in immature interneurons of the subpallial ganglionic eminences (Colombo et al., 2004; Poirier et al., 2004). As these interneurons migrate to their correct position in the cortical plate, they predominantly retain an Arx^+^ identity (Colombo et al., 2004; Poirier et al., 2004). *Ex vivo* experiments have shown that poly-alanine mutations in *ARX* lead to nuclear inclusions and result in increased cell death in mutant-expressing cells; however, these results were obtained in heterologous expression systems that overexpressed *ARX* and have not been corroborated *in vivo* (Nasrallah et al., 2004; Price et al., 2009). During the first two postnatal weeks, GABAergic interneurons normally undergo a wave of apoptosis, reducing their population density by ∼20-30%, with a peak in apoptosis at P7 (Priya et al., 2018; Southwell et al., 2012). Unexpectedly, however, we found that excess cell death in *Arx^(GCG)10+7^* was non-cell autonomous, since mutant Arx^+^ neurons did not contribute to the increase in CC3^+^ cells. One possible explanation is that this cellular phenotype reflects a vulnerable subpopulation of cortical precursor neurons that had once expressed *Arx^(GCG)10+7^* prior to the postnatal window we examined. In the embryonic brain, *Arx* is expressed in neural progenitor cells of the pallial subventricular zone, but with postnatal development and exit from the subventricular niche, these neurons lose *Arx* expression (Colombo et al., 2004; Friocourt et al., 2008). However, we did not find reduced survival of NeuN^+^ neurons that did not express Arx. A study by Beguin and colleagues found no alterations in pyramidal neuron migration and cortical layering in a similar PA1 expansion model or in pyramidal progenitors electroporated with this PA1 expansion mutation; however, they did find aberrant excitatory activity and altered dendritic and synaptic arborization of hippocampal pyramidal neurons, demonstrating a non-cell-autonomous structural defect (Beguin et al., 2013). Although we were unable to definitively identify this population, we speculated that progenitors that had once expressed the *Arx^(GCG)10+7^* may enter aberrant transcriptional programs that later lead to premature cellular demise. To corroborate this, we profiled P7 cortical mRNA expression using Nanostring and the nCounter^®^ mouse Neuropathology panel, but were unable to validate mRNA expression alterations identified in Nanostring analysis using qPCR, with the exception of *Npy* (Table S2, Fig. S3). Further studies are required to specifically determine whether other cortical cells involved in cellular migration that undergo partial or complete apoptosis during postnatal cortical development, such as Cajal-Retzius cells or glia (Wong and Marín, 2019), exhibit altered apoptosis in *Arx^(GCG)10+7^* mutants. However, it is unlikely that Cajal-Retzius cells are preferentially affected as they typically reside in Layer 1 and we found no preferential layer occupied by CC3^+^ cells in *Arx^(GCG)10+7^* mutants (Fig. S1).

### Etiology of elevated apoptosis and potential impact on neonatal cortical development

Acute and chronic inflammation in the immature brain is associated with inappropriate apoptosis and is a risk factor for IS development (Hagberg et al., 2015; Pardo et al., 2014; Shandra et al., 2017). Moreover, the anti-inflammatory peptide hormone ACTH constitutes the frontline medical treatment for IS (Hani and Mikati, 2016). Therefore, we speculated that an innate immune response in the postnatal *Arx^(GCG)10+7^* brain might contribute to increased apoptosis. However, we found no apparent cellular or molecular evidence of inflammation. Interestingly, ACTH(1-24) had no therapeutic effect in *Arx^(GCG)10+7^* mutant mice (Fig. S2A-F). Although the therapeutic action of ACTH may be due to decreasing inflammation in human cases of IS, our data suggest that *Arx^(GCG)10+7^* may be a model of the many clinical cases of ACTH-unresponsive IS. Neither E2 nor ACTH(1-24) targeted exaggerated apoptosis, yet E2 had a therapeutic effect on spasms and electrographic phenotypes in these mice (Olivetti et al., 2014). Thus, the relevance of increased early apoptosis to the epilepsy phenotype, as well as other neurodevelopmental comorbidities in ISSX, remains uncertain.

### Mechanisms of hormonal therapies in ISSX and interneuronopathy models

Interestingly, E2 and ACTH(1-24) increased Arx^+^ density in the motor cortex to a similar degree in *Arx^(GCG)10+7^*, and this effect was regional, since we saw no change in Arx^+^ cell density in the RSA. However, the disparity in clinical efficacy between E2 and ACTH(1-24) suggests that increasing Arx^+^ cells in only some cortical regions is not sufficient to modify disease progression, and other mechanisms driving epileptogenesis, such as disinhibition from mislocalized interneurons, may prevail. The increase in Arx^+^ density could be due to different mechanisms, including upregulated *Arx* expression within an undetected subpopulation of interneurons, or enhanced migration of Arx^+^ cells in the neocortex. Data from *Arx* mouse models suggest that polyalanine expansions in PA1 cause both a decrease in protein levels and affect the ability of *Arx*-expressing interneurons to migrate to the cortex (Lee et al., 2017, 2014; Olivetti and Noebels, 2012). It remains unclear whether this decrease is due to loss of Arx interneurons or reduction of Arx protein by other mechanisms. Previous data from our laboratory showed that neonatal E2 treatment does not affect brain expression of *Arx* mRNA (Olivetti et al., 2014). As E2 treatment has implications for potential clinical trials of ACTH-resistant IS, future work should also determine the effects of upregulating mutant forms of *Arx* such as *Arx^(GCG)10+7^* and whether E2 simply upregulates gene expression or improves outcomes of *Arx^(GCG)10+7^*-expressing cells.

Recent data also suggest that E2 may have neuroprotective effects in interneurons and interneuronopathy models. We previously reported that E2 can increase the density of parvalbumin and neuropeptide Y interneuron subtypes in the somatosensory cortex of *Arx^(GCG)10+7^* (Olivetti et al., 2014). E2 has also been shown to have neuroprotective effects in interneuronopathy models and across species. E2 increases GAD-67^+^ (also known as GAD1^+^) GABAergic interneurons in a rat model of IS (Chachua et al., 2016) and elevates parvalbumin expression in a *Pvalb* KO model of autism with rescue of aberrant phenotypes (Filice et al., 2018). Additionally, premature rabbit neonates treated with E2 exhibited increased cortical Arx and parvalbumin cell densities, upregulation of *Arx* mRNA expression and increased expression of the *Arx* target *Shox2* (Panda et al., 2018). These studies, along with our own, indicate that early treatment with E2 may have a conserved role in elevating Arx*^+^* cells and other GABAergic subtypes. However, to our knowledge, ACTH(1-24) has not yet been shown to have this effect on GABAergic subtypes. Together, these data suggest that there may be shared mechanisms of action between E2 and ACTH and that both hormones may be candidate therapies for interneuronopathies characterized by loss or ectopia of GABAergic interneuron subtypes.

In summary, we have shown a transient increase in developmental brain apoptosis in *Arx^(GCG)10+7^*, as indicated by increases in CC3 expression and TUNEL assay in P7 neocortex. Although the cellular identity of this vulnerable population is unknown, we found that augmented apoptosis in Arx^+^ mutant interneurons is not the cause, indicating that enhanced cell death in *Arx^(GCG)10+7^* neonates is non-cell autonomous. We have also shown that elevated apoptosis is not a result of activation of inflammatory pathways, as we did not find any evidence of astroglial activation, microglial activation or invasion, or elevated cytokine expression in *Arx^(GCG)10+7^* neonates. In addition, ACTH(1-24), a known anti-inflammatory drug and front-line therapy in IS, showed no convincing efficacy in reducing spasms and seizures in *Arx^(GCG)10+7^* mutant mice. However, both ACTH(1-24) and E2 increased Arx^+^ densities in the neocortex, suggesting that these hormones may act on similar pathways in *Arx^(GCG)10+7^*. We conclude that the efficacy of early hormone stimulation in this ISSX model is likely due to direct neuroprotective actions rather than anti-inflammatory activity in immature brain.

## MATERIALS AND METHODS

### *Arx^(GCG)10+7^* mice

*Arx^(GCG)10+7^* mice (*Mus musculus*) were developed previously and described in Price et al. (2009), and maintained on an inbred C57BL/6/129S5/SvEvBrd background (Olivetti et al., 2014; Price et al., 2009). As the *Arx* gene is located on the X-chromosome, only hemizygous *Arx^(GCG)10+7^* male mice and WT male littermates were used in this study. Mice were housed in groups of two to five animals in climate-controlled conditions with 12 h light/dark cycles and allowed access to water and food *ad libitum*. All animal studies conformed to the National Institutes of Health (NIH) Guide for the Care and Use of Laboratory Animals and were approved by the Baylor College of Medicine Institutional Animal Care and Use Committee.

### E2 treatment

E2 (Sigma-Aldrich, E2257) was administered to neonatal mice daily from P3 to P7. E2 administration is described in Olivetti et al. (2014). E2 (40 µg/kg/day) in sterile sesame oil (Sigma-Aldrich, S3547) or sterile sesame oil as a control was administered by daily subcutaneous injection at the same time each day. For treatment at P7, animals were euthanized 2 h after treatment and brains were collected. Litters were treated separately with drug or vehicle and non-littermate controls were used due to drug crossover from mothers to other pups in the litter or skin-to-skin contact between pups.

### Immunohistochemistry and TUNEL assay

Neonatal pups were decapitated at the specified age, and brain tissue was fixed overnight at 4°C in 4% paraformaldehylde and cryoprotected in 30% sucrose. Immunohistochemistry (IHC) was performed on sagittal 20 µm sections taken starting 200 µm from the brain midline. For IHC CC3/Arx studies, six non-consecutive 20-µm sections were used. All other assays utilized three or four non-consecutive 20-µm sections taken starting 200 µm from the brain midline: IBA1/CD68 IHC utilized three slices, GFAP IHC utilized four slices, and NeuN IHC utilized three slices. IHC was performed as described in Olivetti et al. (2014). Slices were blocked using 10% bovine serum albumin in 0.1% Triton X-100, incubated with primary antibody overnight at room temperature, washed and then incubated in fluorophore-conjugated secondary antibody for 1 h. Negative controls were performed by omitting primary antibody. The primary antibodies and concentrations used to perform these experiments include anti-Arx (1:500; UC Davis/NIH Neuromab, clone N411/51), anti-CC3 (1:1000; Cell Signaling Technology, #9661), anti-NeuN (1:1000; EMD Millipore, #ABN78), anti-GFAP (1:500; UC Davis/NIH Neuromab, clone N206A/8), anti-IBA-1 (1:1000; Wako, #019-19741) and anti-CD68 (1:250; Bio-Rad, MCA1957). Secondary antibodies include Alexa-Fluor™ 594 anti-mouse IgG1 (1:1000; Invitrogen, A21125), Alexa-Fluor™ 488 anti-rabbit IgG (1:1000; Invitrogen, A11070), Alexa-Fluor™ 594 anti-rabbit (1:1000; Invitrogen, A11072), and Alexa-Fluor™ 594 anti-mouse IgG2a (1:1000; Invitrogen, A21135). Slides were mounted with VECTASHIELD^®^ Hardset™ Antifade Mounting Medium with 4′,6-diamidino-2-phenylindole (DAPI; H-1500). TUNEL assay was performed on fixed 20-µm sections using the In Situ Cell Death Detection Kit (Roche, 11-684-795-910). Four non-consecutive 20-µm slices starting 200 µm from the brain midline were used for TUNEL assay. Positive controls for TUNEL assay were performed by incubation with 50 U/ml DNAse I (Zymo E1010) for 10 min. Negative controls were performed by omitting terminal transferase enzyme.

### Microscopy and image analysis

All microscopy was performed using a Nikon inverted epifluorescent microscope with an Andor Zyla 4.2 PLUS camera. Nikon NIS Elements Pro software was used to analyze images. All image analysis was performed by an observer blinded to genotype and/or treatment using numerical identifiers. If one or more slices exhibited significant damage, the sample was excluded from analysis. For most experiments, cells were individually counted using the NIS Elements Taxonomy tool. For CC3/Arx IHC, total cells across the entire neocortical plate were quantified. For Arx density analysis, a section 400 µm in width and the depth of the cortical plate were taken from the RSA and the motor cortex of the neocortex. For NeuN analysis, two regions located in the RSA and motor cortex of the neocortex were used. Quantitative analysis was performed with automated cell counting on FIJI (ImageJ version 1.52, JAVA version 1.8.0) by converting images to binary and using the Measure Particles plugin. For GFAP quantification, integrated density of selected regions (RSA, white matter, dentate gyrus and CA1 of hippocampus) was quantified using FIJI. Background integrated density multiplied by the area of section was subtracted from the integrated density of the area of interest to obtain a value for each slice. Four slices were averaged to obtain a mean corrected total fluorescence for each sample. For IBA-1/CD68 IHC, densities of IBA-1^+^ and IBA-1/CD68^+^ co-labeled cells were quantified from the RSA (not shown), motor cortex and hippocampus. For all assays, counts and densities were averaged between six, four or three slices (see ‘Immunohistochemistry and TUNEL assay' section) to obtain an average for one 20-µm slice. For CC3 bin analysis and quantification of cortical depth and area, ImageJ (FIJI version 1.52p) was used to generate six equal bins across the neocortical plate. Values from each bin were averaged between four non-consecutive parasagittal slices between 200 µm and 400 µm from the brain midline to obtain one bin value per sample. Using ImageJ (FIJI version 1.52p), the same four parasagittal slices were also used to measure sagittal area and cortical depth in the RSA and motor cortex (see Fig. S1). Four slices were averaged to obtain one area or depth value per sample.

### mRNA expression profiling using Nanostring and qPCR

Nanostring mRNA expression profiling was conducted using nCounter^®^ mouse Neuropathology Plus Panel, which contains probe sets to query the expression of 800 unique mRNAs (https://www.nanostring.com/products/gene-expression-panels/ncounter-neuropathology-panels). Table S3 contains a list of 29 additional probes that were designed to profile mRNA from Arx targets, and other factors found to be dysregulated in the *Arx^(GCG)7/Y^* model (Kitamura et al., 2009; Mattiske et al., 2016). RNA was extracted from P7 cortical (neocortex and hippocampus) from the left hemisphere using a Qiagen RNeasy Plus kit with genomic DNA eliminator column (74134). Cortices from *Arx^(GCG)10+7^* and WT male littermates were used (six WT and six *Arx^(GCG)10+7^*). The BCM Genome and RNA Profiling Core performed the assay on an nCounter MAX 5 s and RNA quality control using a NanoDrop One and Agilent 2100 Bioanalyzer. Only RNA samples that met strict quality and purity criteria [RNA integrity number (RIN) >8 and no anomalies in Bioanalyzer spectrograph] were submitted for analysis. Output data were analyzed and quality controlled using Nanostring NSolver software (version 4.0.70). Differential expression between genotypes was conducted using a linear regression model in the Advanced Analysis software (version 2.0.115) in nSolver and corrected for batch and litter effects (see https://www.nanostring.com/products/analysis-software/advanced-analysis for more information). mRNAs that were below the detection limit of nCounter MAX 5 s were excluded from analysis. Pathway scoring analysis for cytokines (see Table S1 for list of genes) was also performed in Advanced Analysis software (version 2.0.115) in nSolver, which uses the first principal component of expression data to determine pathway-level information from a group of genes (Tomfohr et al., 2005).

### qPCR of mRNA expression

qPCR was used to profile cytokine mRNA expression and validate expression changes identified in Nanostring analyses. qPCR was performed using 20 ng RNA from separate biological replicate P7 cortices. RNA was extracted and quality was assessed as described above. Real time qPCR assays were performed on an Applied Biosystems Quantstudio 3 using TaqMan Fast Advanced Master Mix (Thermo Fisher Scientific, 4444963) and TaqMan assays (Thermo Fisher Scientific; see Table S4). Data were analyzed using Applied Biosystems Quantstudio Design and Analysis software. Table S4 contains the list of TaqMan probes used for qPCR. qPCR data were analyzed using the 2^−ΔΔCT^ method.

### ACTH(1-24) treatment

ACTH(1-24) (Sigma-Aldrich, A0298) was administered to neonatal mice daily from P3 to P7 or P10. Mice treated from P3 to P7 were used for IHC studies and those treated from P3 to P10 underwent spasms behavioral monitoring or electroencephalogram (EEG) surgery and video-EEG monitoring. As handling pups and time away from mother during spasms monitoring could influence pup development, separate cohorts were treated for EEG experiments. ACTH(1-24) (2 IU/kg/day or 4 IU/kg/day) in sterile saline or sterile saline as a control was administered by daily subcutaneous injection at the same time each day. ACTH(1-24) injections were administered after spasms recordings at ages P7-P10. For IHC, spasms and EEG studies, litters were treated separately with drug or vehicle, and non-littermate controls were used due to potential for drug crossover from mothers to other pups in the litter or skin-to-skin contact between pups.

### Behavioral spasms monitoring and quantification

Spasms were monitored similarly as described in Olivetti et al. (2014). Pups treated with ACTH(1-24) from P3 to P10 were placed in a warmed, compartmentalized container. Each chamber of the container contained a number, which allowed the experimenter to be blinded to treatment. A single pup was placed in a chamber measuring 5.5×7 cm and allowed to habituate for 10 min. Following habituation, pups were recorded with a HD digital video recorder for 50 min. Spasms were monitored daily from P7 to P11 in the light cycle between 17:00 and 19:00 (when pups exhibited wakefulness). ACTH(1-24) subcutaneous injections (P7-P10) were performed after spasms monitoring. Spasm behavioral movements were quantified by a blinded experimenter as previously described in Olivetti et al. (2014). Movements consisting of major truncal flexion, abdominal contractions, and bowing or axial twisting of the body that resulted in pups lying supine were quantified.

### Video EEG

Video EEG was conducted as previously described in Olivetti et al. (2014). Silver wires measuring 2 mm in diameter were implanted in the epidural space to monitor cortical EEG while simultaneously recording video EEG. Mice treated with ACTH(1-24) daily from P3 to P10 were anesthetized using isoflurane and surgically implanted at P35. Video EEG was recorded weekly in 24-h blocks from P45 to P72 in freely moving mice. Total recording time was an average of 89 h per mouse, with at least 72 h of total recording time. Electrographic seizures were quantified by a blinded experimenter as previously described in Olivetti et al. (2014), and seizure frequency was averaged over the total recording time for each mouse. Interictal spikes were quantified from EEG by a blinded experimenter from 4×1-h intervals on different recording days between 15:00 and 17:00. To obtain an average interictal spike frequency, counts from the 4 h were averaged.

### Statistical methods

All statistical analyses were performed using GraphPad Prism 8 (version 8.1.1) software. For all analyses, *N* represents individual biological replicates. Outliers were pre-defined as having values greater than three standard deviations from the group mean and were excluded from analyses. Data exclusion of outliers only applied to IHC analyses. To verify that studies had sufficient sample sizes to detect statistical differences between means, power analyses were conducted with a power of 80% for all analyses. If studies were deemed to be underpowered based on power analyses, additional cohort(s) were added to increase *N*. Prior to applying all parametric statistical tests that assume Gaussian distribution, the Shapiro–Wilk normality test was conducted to statistically determine if data distributed on a normal curve. If data failed this normality test, the appropriate non-parametric statistical analyses were used. Unless otherwise noted, all graphs are displayed as the genotype mean±s.e.m. For analyses that include multiple comparisons, appropriate post-hoc analyses were used that incorporate *P*-value corrections for multiple hypotheses testing. Statistical analyses and post-hoc analyses used in each assay are described in figure legends.

## Supplementary Material

Supplementary information
