# Supplementary material for: Arx expansion mutation perturbs cortical development by augmenting apoptosis without activating innate immunity in a mouse model of X-linked infantile spasms syndrome
Source: Dis Model Mech. 2020 Mar 30;13(3):dmm042515. doi: 10.1242/dmm.042515 (PMC7132796; doi:10.1242/dmm.042515)
Supplement: Supplementary information [file dmm-13-042515-s1.pdf]

Supplemental Information

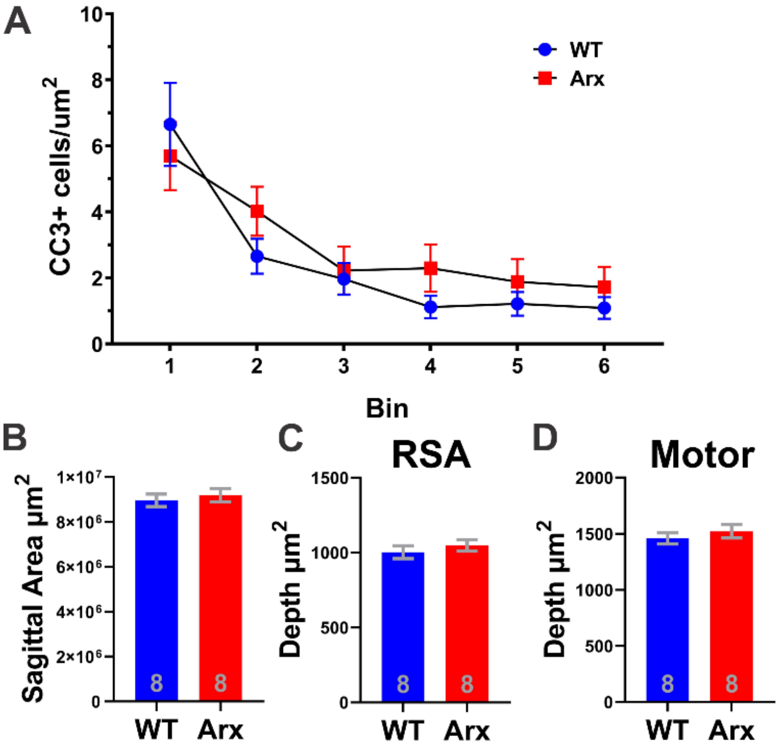

Fig. S1. *Arx*$^{(GCG)10+7}$ mutants do not exhibit differences in CC3 distribution or neocortical size compared to WT littermates at P7. (**A**) Distribution of CC3+ density in Arx mutants and WT littermates in 6 laminar bins (1 most superficial, 6 most deep bin). There was no difference between genotypes (F(1,96)=1.681, P=0.1979), a significant effect of bin (F(5,96)=13.81, P<0.0001), but no interaction between bin and genotype (F(5,96)=0.7028, P=0.6226) (Two Way ANOVA). N=10 WT, 8 Arx. Mean and SEM are displayed. (**B**) Area (µm^2) of WT and *Arx*$^{(GCG)10+7}$ mutant sagittal slices (P=0.5812, Unpaired t test). (**C-D**) Depth (µm) of two different cortical regions showing no difference between genotypes: (C) RSA cortex (P=0.4316, Unpaired t test) and (D) Motor cortex (P=0.4321, Unpaired t test) cortex. N=8 WT, 8 Arx. Mean and SEM are displayed.

Disease Models & Mechanisms • Supplementary information

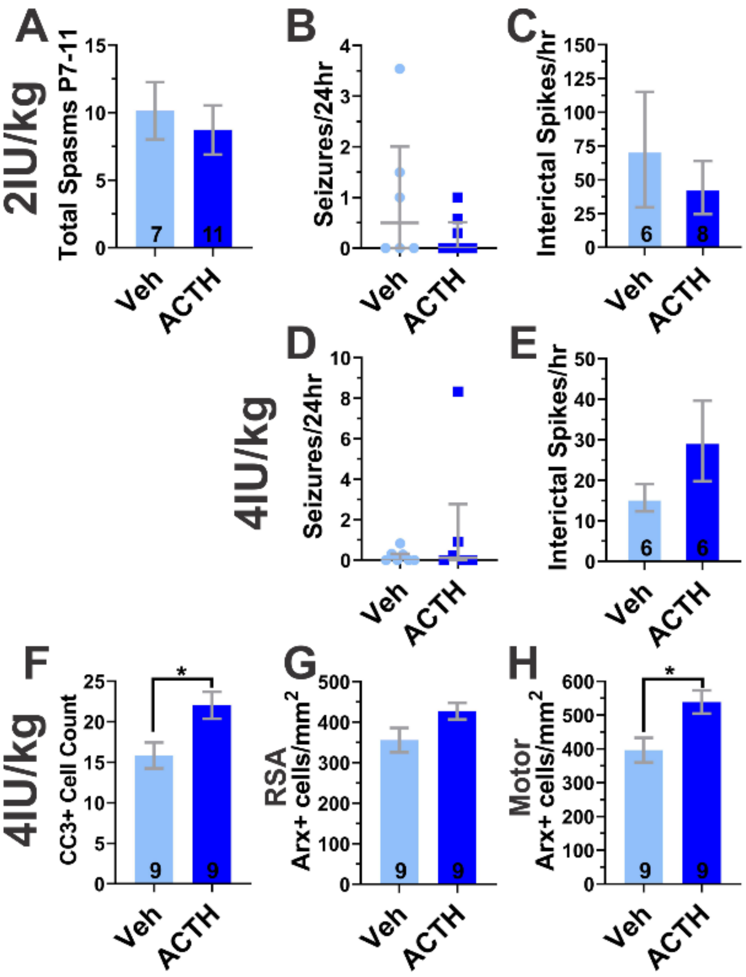

Fig. S2. Neonatal ACTH(1-24) treatment modifies cellular pathology but has no effect on neonatal spasms and electrographic phenotypes in adult *Arx*$^{(GCG)10+7}$. Effect of daily P3-10 ACTH(1-24) treatment at (**A-C**) 2IU/kg/day and (**D-H**) 4IU/kg/day compared to saline vehicle (Veh) control injections. (**A**) Average cumulative P7-11 spasms (p=0.6253, Unpaired T Test). (**B**&**D**) Average daily seizure frequency (seizures/24hr) from weekly P45-72 (average of 89 hours of total recording per mouse) video EEG recordings following neonatal 2IU/kg/day or 4IU/kg/day ACTH(1-24) treatment. (B) 2IU/kg/day ACTH(1-24) treatment (p=0.3287, Mann Whitney Test) (N=6 Veh, 8 ACTH(1-24)). (D) 4IU/kg/day ACTH(1-24) treatment (p=0.8701, Mann Whitney Test) (N=6 Veh, 6 ACTH(1-24)). (**C&E**) Average interictal spike frequency (interictal spikes/hr) following neonatal 2IU/kg/day or 4IU/kg/day ACTH(1-24) treatment. (C)

2IU/kg/day ACTH(1-24) treatment (p= 0.4908, Mann Whitney Test). (E) 4IU/kg/day ACTH(1-24) treatment (p= 0.3939, Mann Whitney Test). (**F-H**) Effect of P3-7 4IU/kg/day ACTH(1-24) treatment compared to vehicle (Veh) treatment (saline) on CC3+ cell counts and Arx+ cell density in *Arx*$^{(GCG)10+7}$ at P7. (**F**) Mean CC3+ counts in neocortex with ACTH(1-24) treatment (p= 0.0166, Unpaired T Test) (**G-H**) Mean Arx+ cell density (Arx+ cells/mm^2) with ACTH(1-24) treatment in (G) retrosplenial agranular (RSA) region (p=0.0673, Unpaired T Test) and (H) motor cortex (p=0.0117, Unpaired T Test). Mean and SEM are displayed. With the exception of B&D, all Ns are displayed on graph.

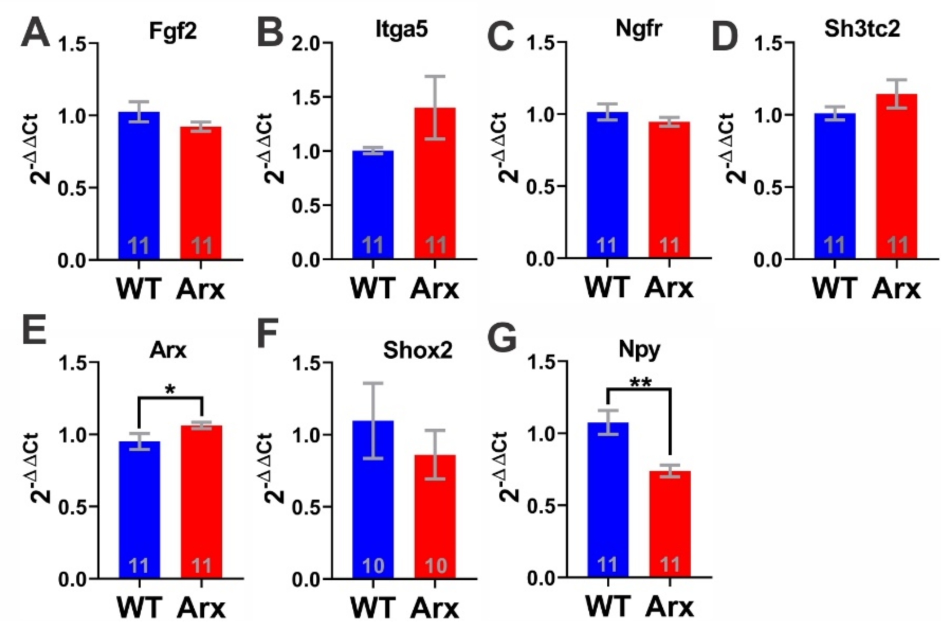

Fig. S3. Validation of Nanostring candidates using qPCR did not reveal mRNA expression changes in *Fgf2*, *Itga5*, *Ngfr*, *Sh3tc2*, and *Shox2* but did reveal changes in *Arx* and *Npy* expression in *Arx*$^{(GCG)10+7}$ P7 cortex compared to WT littermates. (A-G) Average expression ($2^{-\Delta\Delta Ct}$) of each mRNA candidate is displayed. (**A**) *Fgf2* (p=0.1330, Mann Whitney Test) (**B**) *Itga5* (p=0.4385, Mann Whitney Test) (**C**) *Ngfr* (p=0.3316, Mann Whitney Test) (**D**) *Sh3tc2* (p=0.3653, Mann Whitney Test) (**E**) *Arx* (p=0.0400, Mann Whitney Test) (**F**) *Shox2* (p=0.6842, Mann Whitney Test) (**G**) *Npy* (p=0.0019, Mann Whitney Test). Mean and SEMs are displayed. Ns are displayed on graph.

Disease Models & Mechanisms • Supplementary information

Ccl12	*Cx3cr1*	*Fasl*	*Il4ra*	*Prl*
Ccl5	*Cxcl10*	*Flt1*	*Il6*	*Tgfb1*
Ccr2	*Cxcl11*	*Flt4*	*Il6ra*	*Tgfbr2*
Ccr5	*Cxcl12*	*Hgf*	*Inhbb*	*Tnf*
Cd40	*Cxcl16*	*Il10*	*Lif*	*Tnfrsf10b*
Cntf	*Cxcr4*	*Il10ra*	*Ltbr*	*Tnfrsf11b*
Csf1	*Egf*	*Il13ra1*	*Ngfr*	*Tnfrsf12a*
Csf1r	*Egfr*	*Il15ra*	*Osmr*	*Tnfrsf1a*
Csf2rb	*Epo*	*Il1b*	*Pdgfrb*	*Tnfrsf1b*
Cx3cl1	*Fas*	*Il1r1*	*Plekho2*	*Vegfa*

Table S1.

List of 50 cytokines and cytokine-related mRNA's from NCounter mouse Neuropathology panel used in Nanostring pathway analysis (See Fig. 5A).

mRNA	Correction Set	Uncorrected P-value	Corrected P-Value	Log2 fold change	Linear fold change
Ngfr-mRNA	Overlapping	0.00138	1	-1.110	0.465
Sh3tc2-mRNA	Overlapping	0.00707	1	-0.628	0.647
Fgf2-mRNA	Litter Correction	0.00454	1	-0.325	0.798
Itga5-mRNA	Overlapping	0.00205	1	-0.319	0.801
Hdac7-mRNA	Overlapping	0.00135	1	-0.173	0.887
Npy-mRNA	Batch Correction	0.00462	1	-0.171	0.888
Sirt1-mRNA	Batch Correction	0.00681	1	-0.162	0.894
Vegfa-mRNA	Overlapping	0.00583	1	-0.141	0.907
Atf4-mRNA	Overlapping	0.00878	1	-0.118	0.921
Htr1a-mRNA	Batch Correction	0.00797	1	-0.115	0.923
Acaa1a-mRNA	Batch Correction	0.00493	1	-0.093	0.937
Pnkd-mRNA	Batch Correction	0.00945	1	-0.094	0.937
Ran-mRNA	Overlapping	0.00594	1	-0.092	0.938
Ap4s1-mRNA	Overlapping	0.00354	1	-0.081	0.945
Ikbkb-mRNA	Batch Correction	0.00959	1	0.080	1.06

Table S2. Differentially expressed mRNA candidates between WT and *Arx*$^{(GCG)10+7}$ using Nanostring. Data was corrected for batch and litter effects and 15 differentially expressed candidates were chosen based on uncorrected P-value less than 0.01. Highlighted candidates were chosen for validation by qRT-PCR.

Disease Models & Mechanisms • Supplementary information

Gene Name	Accession ID	Probe Target Sequence
**Arx**	NM_007492.3	TACTGAAAATAACCCGGGAGGGGTTCTCCCCAATATGCTTAGTATTGAAGTTTAAAAATTAAAAGCCCAACCTTCTCTTCCTCCGGATACCCCACTTAGC
**Birc6**	NM_007566.3	TCGGCGCTCAGCGCTAAACCAGGTGGACAGGTGAAATGTCAGTATATTTCTGCTGTGGATAAAGTGATATTTGTGGATGATTACGCCGTGGGCTGTAGGA
**Csrnp3**	NM_178634.2	AAAGGACTTTGGGTTTGTCATGCTATTGAAAGCAGTAATGGGGGCTGACTACTCCTTTTGGTTGACCGGTGGGGCTAATCTGGTGGGAATTCTGTGGTAA
**Cxcr5**	NM_007551.2	ACCGTGCCTTTCTCTTAACCAAGCAGAAAGCTGAAACCGATCTACTTTAGGTAGCTGTCTGGTTCCAACCTAACCAGCATTGGGTCAGCCCCATGTTACT
**Drap1**	NM_024176.1	TGCCAAAACCATGACCACGTCCCACCTGAAGCAGTGCATTGAACTCGAGCAGCAGTTTGACTTCTTGAAAGACTTGGTGGCATCAGTGCCTGACATGCAG
**Ebf1**	NM_007897.2	CATAGGGGACAATTTCTTTGATGGGTTACAGGTCATATTCGGTACCATGCTGGTCTGGAGTGAGTTGATAACTCCTCATGCCATCCGAGTTCAGACACCT
**Endog**	NM_007931.1	GGAACAACCTTGAGAGGTACAGCCGCAGCTTGACGCGAACTTACCAAAATGTCTATGTCTGCACGGGGCCGCTTTTCCTGCCCAGGACCGAGGCTGATGG
**Erbb4**	NM_010154.1	TCCTGGTGATCATGGCTTTGACATTTGCTGTCTATGTCAGAAGAAAGAGCATCAAAAAGAAACGTGCTTTGAGGAGATTCCTGGAGACAGAGCTGGTAGA
**Esr1**	NM_007956.4	ACTCGCTACTGTGCCGTGTGCAATGACTATGCCTCTGGCTACCATTATGGGGTCTGGTCCTGCGAAGGCTGCAAGGCTTTCTTTAAGAGAAGCATTCAAG
**Esr2**	NM_010157.3	TGCTTCTTATAACTGTAGCCAGTCCATCCTACCCTTGGAGCATGGTCCCATCTATATCCCTTCCTCCTATGTAGAGAGCCGTCACGAATACTCAGCCATG
**Ghrh**	NM_010285.2	GGATGCAGCGACACGTAGATGCCATCTTCACCACCAACTACAGGAAACTCCTGAGCCAGCTGTATGCCCGGAAAGTGATCCAGGACATCATGAACAAGCA
**Gnmt**	NM_010321.1	GGGTCATTGAAGAAGCCAACTGGTTGACGCTGGACAAAGATGTGCTTTCAGGAGATGGCTTTGATGCTGTCATCTGCCTTGGGAACAGTTTTGCTCACTT
**Hdac4**	NM_207225.1	AGACGCTGAGTACTTGGCAGCCTTCAGAACGGTGGTTATGCCTATCGCAAATGAGTTTGCCCCAGATGTGGTACTGGTGTCATCGGGCTTCGATGCTGTG
**Hipk2**	NM_010433.2	AGGAAGCAAGAAAGTACATTTTCAACTGTTTGGATGATATGGCCCAGGTGAACATGACAACAGATTTGGAAGGGAGTGACATGTTAGTAGAGAAGGCAGA
**Kcnma1**	NM_001253361.1	CGCATGTGGTGGGCTTTCTTGGCCTCCTCCATGGTGACTTTCTTCGGGGGCCTCTTCATCATCTTGCTCTGGCGGACGCTCAAGTACCTGTGGACCGTTT
**Kif26b**	NM_001161665.1	CATGATCGGAAGGGACGACTCTATGCAGAACCTGGGCATTATTCCATGTGCTATTTCTTGGCTGTTCAAGCTTATCAATGAGCGCAAAGAGAAGACGGGG

Disease Models & Mechanisms • Supplementary information

**Mef2c**	NM_001170 537.1	TTCTACTACTAAAGGTATCAATGGAACATGAAGACG AGTATTTAGGCAGAAGCAAAACAGGAAACCATCCTT ACAAACATGCTTACCTGCACATCTGTTT
**Msx2**	NM_013601. 2	CTACCCCTTCCATAGACCTGTGCTCCCCATCCCGC CTGTTGGACTCTATGCCACGCCGGTTGGATATGGC ATGTACCATCTATCCTAAGGAAGACCAGAT
**Myt1l**	NM_001093 775.1	ATCAGTGACAGAAGTTATGCTGAGGGGATGTCACA GCAGGACAGTAGAAATATGAACTATGTCATGCTAG GGAAGCCCATGAACAATGGACTCATGGAGA
**Ndufa13**	NM_023312. 2	CAGATTCTCCGGGAAAACCTGGAGGAGGAAGCCAT CATCATGAAGGATGTGCCCAACTGGAAGGTGGGC GAGTCTGTGTTCCATACCACACGATGGGTGC
**Pcdhga5**	NM_033588. 4	CAGTACTTTCGAGCACCGTCCATATCAGCGTGACC GTTCTTGATGCAAACGATAACGCACCCTTGTTTACC CAAAGCGAATATAGGGTGAGTGTTCCGGA
**Pde3a**	NM_018779. 1	ATATAGGAAGAAAATGTGGCCGTATTCTGAGCCAG GTATCATACAGACTGTTTGAAGACATGGGGCTCTTT GAAGCCTTTAAAATCCCGGTTAGGGAGTT
**Perp**	NM_022032. 4	CTTCACGATAACCCTGCTGTTAATTACATCTATAAC TGGGCCTATGGCTTCGGATGGGCGGCCACCATCAT CTTGATTGGTTGTTCCTTCTTCTTCTGCT
**Prdx5**	NM_012021. 2	AAAGAAGGTGAACTTGGCAGAGCTGTTCAAGGGCA AGAAAGGTGTTTTGTTTGGAGTCCCTGGGGCATTT ACACCTGGCTGTTCTAAGACCCACCTGCCT
**Samd5**	NM_177271. 3	GATGAGATGCGGAAGACTGGAAAGGGGCGTATTTA GGACCTTCTTTCACAAAGGGAACTGGATGGTGACT CTGCAAACACTTATCAGCTTAACGTTTTCT
**Sfn**	NM_018754. 2	AATCTGATTTGGTAATCCAAGACGCTCCTGCAATGC AGCCAGCCCTGAACTGCAGGGGGCAGTCTGGAGC CGAAAGGTGCCTTTGCAGGTGGGACCTGCG
**Shox2**	NM_013665. 1	ATTTTACCCTGGAACAACTCAACGAGCTGGAGAGG CTTTTCGATGAGACCCACTATCCAGACGCTTTCATG CGCGAGGAATTGAGCCAGCGACTGGGGCT
**Twist1**	NM_011658. 2	AATGGACAGTCTAGAGACTCTGGAGCTGGATAACT AAAAATAAATCTATATGACAAAGATTTTCATGGAAAT TAGAAGAGCAGAGACCAAATTCACAAGA
**Zfp369**	NM_178364. 5	GTTACAAGGTTCATCTGCACAAAATCACCAGATGG GGTCTAGGGCAGGAAGAGCCAGGGACAACAGCAT CTTAACACATGTAAAAATTCACCAGAAAGGC

Table S3

List of 29 additional mRNA probes added to the Nanostring Mouse Neuropathology panel.

Disease Models & Mechanisms • Supplementary information

Probe	TaqMan™ Assay ID	Use
Lars	Mm00506560_m1	Normalizer/Housekeeping
Tnf	Mm00443258_m1	Target
Il6	Mm00446190_m1	Target
Il1b	Mm00434228_m1	Target
Fgf2	Mm00433287_m1	Target
Itga5	Mm00439797_m1	Target
Ngfr	Mm00446294_m1	Target
Sh3tc2	Mm01261770_m1	Target
Arx	Mm00545903_m1	Target
Shox2	Mm00443183_m1	Target
Npy	Mm00445771_m1	Target

Table S4

TaqMan™ Gene Expression Assay ID's used for qPCR validation. *Lars* mRNA expression was used as a normalization/housekeeping gene as it was computationally identified by Nanostring assay as having adequate expression and low variability between samples.

Disease Models & Mechanisms • Supplementary information
